# Human Health Risk Assessment associated with contaminants in the finest fraction of sidewalk dust collected in proximity to trafficked roads

**DOI:** 10.1038/s41598-019-52815-0

**Published:** 2019-11-08

**Authors:** Ewa Adamiec, Elżbieta Jarosz-Krzemińska

**Affiliations:** AGH University of Science and Technology, 30 Mickiewicza Av., 30-059 Kraków, Poland

**Keywords:** Environmental impact, Risk factors

## Abstract

The objective of the study was to determine concentration of metals in sidewalk dust collected in close vicinity to heavily congested roads in Poland in order to assess non-carcinogenic and carcinogenic health risk for both children and adults associated with the ingestion, dermal contact and inhalation of sidewalk dust. Results revealed that sidewalk dust from Warsaw, Krakow, Wroclaw and Opole is heavily contaminated especially with Sb, Se, Cd, Cu, Zn, Pb, considered as indicators of traffic emission. Hazardous indices determined for different exposure pathways indicated that the greatest health risk for both children and adults is associated with the ingestion of sidewalk dust. Carcinogenic risk associated with the ingestion of sidewalk dust by children, calculated for As, Cd, Ni and Pb exceeded safe level of 1 × 10^−4^ in all cities except for Warsaw. Non-carcinogenic risk of ingestion for children was two orders of magnitude higher than dermal risk and four to five orders of magnitude higher than risk of inhalation. Non-carcinogenic risk associated with the ingestion of sidewalk dust by adults is comparable with dermal contact risk and five orders of magnitude higher when inhalation risk.

## Introduction

Air pollution related to road transport is considered the main environmental risk factor, accountable for premature deaths worldwide^1–3^. It is estimated that about 400 thousand people die prematurely each year in Europe^4,5^ as a result of respiratory, cardiovascular diseases^6–8^ and various types of cancers, in particular lung cancer^9–11^. It is believed that transport-related air pollution results only from the effect of incomplete fuel combustion, whereas in fact non-exhaust particle emission prevails, generated by abrasion of brake linings and clutch plates, corrosion of car body and road infrastructure and degradation of road sidewalk^12–17^. According to data from Polish Statistic Office (GUS)^18^^,^ in Poland in 2014 non-exhaust sources has accounted for up to 78.4% of the total dust pollution from transport sources. Some authors^19^ predict even that the share of non-exhaust particles will constantly grow and by the end of 2020 it will account on average 90% of transport related pollution.

Advanced research on exhaust particulate emissions and their impact on the environment and human health has been conducted since the 70-ties of the last century. Consequently, it has forced increased legislative measures and significant technological improvements, thus contributing to the reduction of particulate emission from combustion engines^17,20,21^. At the same time, research on non-exhaust sources have only begun to appear^5^ and no legislative measures or technological improvements aiming at non-exhaust emission reduction have been undertaken. Even that non-exhaust sources have not been yet adequately investigated^21^, preliminary research on this subject shows that their health impact is most relevant^5^. The latest research^10^ has revealed much higher oxidation stress potential of particles from spent brake linings than diesel exhaust emission, tyres or road dust. *In vitro* toxicity examinations, carried out on animals, has confirmed that the chemical constitution of road dust particles plays an important role in their toxic, genotoxic and carcinogenic mechanisms, however these mechanisms have not been unambiguously explained.

Some authors reports^22,23^ that non-exhaust particles (e.g. from brake wear) rather than geogenic particles are subject to re-suspension in road dust due to smaller size and thus they are more oral bioaccessible and they may cause serious potential health risk for inhabitants when airborne. Since as much as 50% of non-exhaust particles can undergo resuspension^20,22,24^ and be deposited on road surface, sidewalks or nearby, they should also be considered as a secondary source of pollution. Moreover considering the fact that road dust is enriched with heavy metals especially in close vicinity to heavily congested and stop/start areas of the cities^25,26^, it seems therefore reasonable to assess potential health risk associated with the exposition of inhabitants of big, congested cities to sidewalk dust.

The main objective of the study was (1) to determine concentration of metals in sidewalk dust collected in close vicinity to main roads in four big cities of Poland and to calculate its pollution indices (PI); (2) to assess non-carcinogenic and carcinogenic health risk for both children and adults associated with the ingestion, dermal contact and inhalation of sidewalk dust by calculating hazard quotient (HQ) and hazard index (HI) for each metal and each exposition pathway.

## Materials and Methods

### Samples locations and sampling collection

Total of 56 samples of sidewalk dust were collected from four big cities of Poland, that is from Warszawa - the capital of Poland, Krakow - second biggest city with 769 498 inhabitants, Wroclaw- the fourth biggest city of Poland with the population of 639 258 and Opole- much smaller city, estimated as 26^th^ biggest, with 128 224 inhabitants. In Warszawa live approximately 1.770 million residents, however the whole great metropolitan area of Warszawa is the 8^th^ most populous capital city in EU with 3.1 mln residents. It is estimated that daily approximately 690 000 cars is entering Warszawa, in Kraków 246 000 cars, in Wroclaw 238 000 and in Opole 42 000 cars respectively. From all cities of Poland Krakow is regarded as the most congested city, mostly due to unfavorable road grid.

In Warszawa samples of sidewalk dust were collected from two locations, from Flotyli Wislanej Boulevard (52°23,5084′N 21°03,454′E), Zbigniew Religa Boulevard (52°26,2333′N 21°00,0710′E). In Kraków samples were collected from Czerwinski Boulevard (50°05,3691′N 19°92,9135′E) and along Nowohucka St. (50°05,4967′N 19°99,6372′E). In Wroclaw samples were collected from sidewalk along Jednosci Narodowej St. (51°12,8715′N 17°05,4807′E) and Wejcherowska St. (51°13,2225′N 16°98,9751′E). In Opole samples were collected along Krapkowicka St. (50°66,7909′N 17°90,6588′E) and Nysy Łużyckiej St. (50°40,367′N 17°54,860′E).

All samples locations were situated in proximity to main roads. After sprinkling with ultra pure water dust was swept from sidewalks (rectangle 4 m x 1 m) using brush, then placed in Ziploc bag and transported to the laboratory.

### Methods

Metals were extracted from fine fraction (<20 µm) of the sidewalk dust samples using *aqua regia*, according to microwave digestion protocol 3050A^27^. Concentrations of the following metals Al, As, Ba, Cd, Co, Cr, Cs, Cu, Fe, Mg, Mn, Mo, Ni, Pb, Rb, S, Sb, Se, Si, Sn, Sr, Ti, U, V, W, Zn and Zr were there determined via ICP-MS (ELAN 6100 PerkinElmer) according to USEPA method 6020B^28^_._ In order to obtain unambiguous and unbiased results of ICP-MS analysis, reagent blanks as well as certified international reference materials (METRANAL^TM^ 32, ERM-CZ120 as well as SRM 1848a) were used.

The concentration of Cu, Pb, Zn, Cr, Mn and Cd in sidewalk dust was determined using ICP-MS and/or ICP-OES as well as AAS methods.

Concentrations were then compared with the World Average Shale Value (AVE), considered as a geochemical background values (BV) for the fraction <20 µm according to Turekian & Wedepohl^29^. Furthermore PI (pollution index) was calculated according to the Eq. 1:

1 $${PI}=\frac{{Ci}}{{Ni}}$$

where Ci refers to metal concentration and Ni- is the geochemical background value according to Turekian & Wedepohl^29^^.^

Phase composition of sidewalk dust were carried out via X-ray Diffractometers (SmartLab (9 kW) RIGAKU with a high temperature camera HTK 1200, Miniflex 600 RIGAKU, APD X’Pert Pw3020 PHILIPS).The interplanar distances obtained from the X-ray patterns were used for identifying crystalline phases based on the data of the ICDD (International Centre for Diffraction Data) catalogue and the XRAYAN software.

In order to assess non-carcinogenic risk for children and adults an average daily intake dose of deleterious substances and exposure through ingestion (ADD_ing_), dermal contact (ADD_derm_) and inhalation (ADD_inh_) were calculated for sidewalk dust according to USEPA^30^ based on exposure factors provided by Regional Screening Levels (RSLs) - Generic tables^31^ and data as well as formulas provided by authors^25,32–34,36^ (Table 1) (Eqs 2, 3 and 4).

2 $${ADDing}=\frac{({Ci}\times {IngR}\times {EF}\times {ED}\times {CF})}{({BW}\times {AT})}$$

3 $${ADDinh}=\frac{({Ci}\times {InhR}\times {EF}\times {ED}\,)}{({PEF}\times {BW}\times {AT})}$$

4 $${ADDderm}=\frac{({Ci}\times {SL}\times {SA}\times {ABS}\times {EF}\times {ED}\times {CF})}{({BW}\times {AT})}$$

**Table 1:** Parameters necessary for the assessment of health risk^25,31,34,35^.

Parameters	Descriptions	Children	Adults
BW	average body weight (kg)	15	70
EF	exposure frequency (days)	180	180
ED	expousure duration (years)	6	24
IngR	Ingestion rate for soil expressed in mg/day	100	50
SL	Skin adherence factor mg/(cm^2^*h)	0.2	0.2
SA	Skin surface area(cm^2^)	2800	5700
CF	conversion factor (kg/mg)	10^−6^	10^−6^
ABS	Dermal absorption factor (unitless)	10^−3^	10^−3^
PEF	Partical emission factor (m^3^/kg)	1.316 × 10^9^	1.316 × 10^9^
InhR	Inhalation rate (10m^3^/day)	10	20
AT	Average time (EDx365days)	6 × 365	24 × 365

Potential non-carcinogenic risk related to specific metals were then assessed for each pathway using hazard quotient (HQ) according to the Eqs 5, 6 and 7:

5 $${HQing}=\frac{{ADDing}}{RfDing\,}$$

6 $${HQderm}=\frac{{ADDderm}}{({RfDderm}\times {GIABS})}$$

7 $${HQinh}=\frac{{ADDinh}}{{RfDinh}\times 1000\,\mu {g}\times {m}{{g}}^{-1}}$$

Where:

RfD_ing_- oral reference dose (mg/kg per day) obtained by Regional Screening Levels (RSLs)- Generic tables^31^

RfD_inh_- inhalation reference concentration (mg/m^3^)

RfD_derm_-dermal reference dose (mg/kg per day)

GIABS- gastrointestinal absorption factor

If HQ exceeds threshold value of 1, potential adverse health effect may occur. The greater the value of HQ above unity, the greater the level of concern^34,37^.

Moreover hazard index (HI) as a sum of individual HQ was calculated in order to assess the overall potential of non-carcinogenic effects posed by more than one deleterious substances.

If HI < 1 then no significant risk occurs, but when HI > 1 chronic risk more likely occurs.

Carcinogenic risk for individual pathways were calculated according to Eqs 8,9 and 10 and as total carcinogenic risk was calculated was according to Eq. 11:

8 $${CRing}={ADDing}\times {SF}$$

9 $${CRinh}={ADDinh}\times {IUR}$$

10 $${CRderm}={ADDderm}\times (\frac{{SFo}}{{GIABS}})$$

Where:

SF- slope factor

SF_o_ – oral slope factor ((mg·kg^−1^·day^−1^)^−1^)

IUR – inhalation unit risk (µg·m^−3^)^−1^

11 $$CRisk=CRing+Crinch+CRderm$$

If the risk is higher than threshold value of 10^−4^ –10^−6^, the risk is considered as unacceptable according to US EPA^38^.

## Results

### Geochemical composition of sidewalk dust

**Figure 1:**
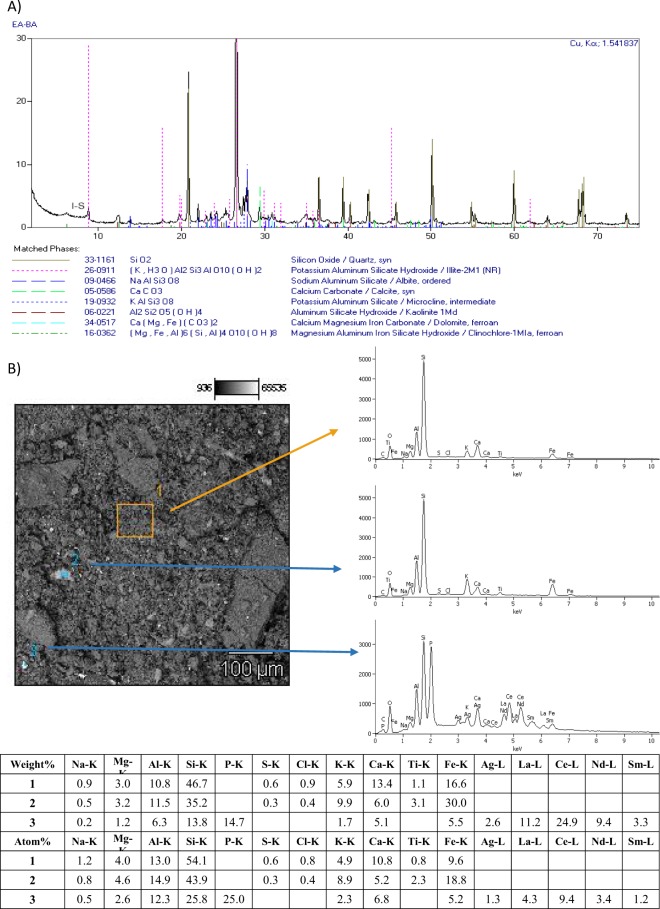
Phase composition (**A**) and SEM – EDS analysis (**B**) of sidewalk dust from Krakow.

Phase analysis conducted via XRD method (Fig. 1A) revealed that sidewalk dust is predominantly comprised of quartz, then to a lesser extent it consists of minerals such as potassium feldspars (microcline), plagioclases (albite), chlorite (clinochlore), calcite and dolomite, as well as a group of clay minerals (such as smectite, illite, kaolinite and mica).

Results of SEM-EDS analysis (Fig. 1B) confirmed the presence of quartz and aluminum silicates as main constituents of sidewalk dust, however other compounds such as titanates or elements like Ag and some Rare Earth Elements (REE) such as La, Ce, Nd and Sm were also detected. These above mentioned secondary constituents of sidewalk dust could be of probable anthropogenic origin since alkali metal titanates (e.g. potassium and sodium titanates) are commonly used as inorganic fillers, which promotes stability of the friction coefficient^39^. Moreover REE in sidewalk dust could be sourced from various components of hybrid electric vehicles (HEVs), since lanthanum is used in batteries, catalyst and lenses, cerium is currently used as fuel additive, catalyst or optical polish, neodymium and samarium are commonly used in lasers, magnets or in car computers and LCD screens^40^.

### Concentration of heavy metals in sidewalk dust

Concentration of metals in sidewalk dust from Warszawa, Krakow, Wroclaw and Opole are presented in Table 2.

**Table 2:** Statistical parameters of traffic-related elements in sidewalk dust.

Elements (mg/kg)	Krakow				Opole				Wroclaw				Warszawa			
	min	max	mean	std	min	max	mean	std	min	max	mean	std	min	max	mean	std
Al	24700	37000	28000	4490	26000	46000	33000	3679	21000	32000	27000	3430	19000	27000	24000	9160
As	14.2	23.5	18.6	1.25	2.37	7.89	6.02	1.26	1.19	17.9	7.50	3.25	2.90	12.7	6.56	3.48
Ba	458	727	557	105	416	531	463	4.23	399	833	620	126	517	672	604	247
Cd	4.38	6.14	5.03	0.567	1.63	2.29	1.95	0.204	1.24	21.3	6.54	3.71	0.980	1.70	1.32	0.537
Co	17.1	34.6	21.3	5.28	23.3	40.5	30.2	3.57	19.7	39.8	32.0	3.7	18.7	25.1	22.2	8.67
Cr	138	176	164	19.6	132	217	166	19.2	141	300	241	77.1	183	242	211	89.6
Cs	824	1300	1000	186	730	939	822	51.1	709	1433	1100	226	932	1220	1070	422
Cu	301	992	573	279	281	472	347	53.8	365	659	448	86.2	560	1130	831	372
Fe	50100	64700	59300	5350	43900	67900	54200	8307	54100	67200	59400	10400	66100	103300	81800	33400
Mn	1710	1960	1850	97.1	939	1670	1200	295	978	1310	1090	125	1150	1380	1260	498
Mo	8.10	16.4	12.5	2.55	7.13	11.7	9.49	0.909	7.34	17.6	10.8	3.71	16.0	29.3	22.4	9.98
Ni	58.8	76.0	67.9	2.62	92.2	151	116	16.8	67.9	300	206	76.1	77.7	109	93.4	37.0
Pb	238	426	311	72.1	180	270	216	51.5	146	280	193	40.6	162	309	216	94.2
Sb	50.1	158	102	36.4	37.6	50.2	43.0	4.27	36.5	128	60.7	37.9	108	185	136	52.2
Se	178	358	261	65.0	195	409	265	38.3	124	281	221	51.5	241	372	278	118
Sn	31.6	54.5	42.6	9.21	27.1	43.8	33.0	4.58	37.3	63.4	46.8	10.9	48.2	73.5	59.5	25.4
Sr	191	314	251	52.8	273	344	322	26.9	216	439	309	91.0	241	300	259	102
Ti	1180	1660	1320	185	1750	3520	2380	425	1200	2860	2090	435	984	1530	1410	553
W	55.5	125	80.8	17.6	38.9	45.7	42.3	3.57	41.6	122	67.6	37,0	46.8	85.1	64.3	25.7
Zn	1780	4400	2690	1000	1230	1460	1330	125	1230	4180	2140	868	1460	2710	1990	810
Zr	2.53	13.2	6.55	1.43	3.04	11.6	5.57	2.18	1.97	9.48	5.97	2.38	3.91	7.07	6.00	2.32

The concentration of metals considered as key tracers of non-exhaust emission in sidewalk dust collected from all cities were significantly elevated or even extreme when compared to background values. Pollution indices (PI) have exceeded value of 1 (unpolluted) with respect to most of the analyzed metals thus confirming contamination of the sidewalk dust with: As, Cd, Co, Cr, Cu, Fe, Mg, Mn, Mo, Pb, Sb, Sn, Se and Zn. The highest PI indices were determined with respect to Sn, Sb, W, Zn, Cd, Pb and Cu in all sidewalk dust samples. Concentration of antimony (Sb) in sidewalk dust has exceeded background values 91 times in Warszawa (PI 91), 68 times in Krakow (PI 68), 40 times in Wroclaw (PI 40) and 29 times in Opole (PI 29). Sidewalk dust was also heavily contaminated with tungsten W in Krakow on average 80.8 mg/kg (PI 45), Warszawa 64.3 mg/kg (PI 36), 67.6 mg/kg in Wroclaw (PI 38) and 42.3 mg/kg in Opole (PI 23). Moreover an average concentration of Zn was ranging from 1330 mg/kg in Opole (PI 14) up to 2686 mg/kg in Krakow (PI 28). Mean concentration of Cd was as high as 6.54 mg/kg in Wroclaw, thus exceeding almost 22 times the background value of 0.30 mg/kg (PI 22). Cadmium pollution indices determined in other cities were also very high and were as follows: in Krakow PI 16 (mean concentration 5.03 mg/kg), in Opole PI 6 (mean concentration 1.95 mg/kg) and in Warszawa PI 4 (1.32 mg/kg od Cd) respectively. Sidewalk dust was also enriched with lead, exceeding multiple times the background values in all investigated cities (PI 15 in Krakow, PI 11 in both Opole and Warszawa and finally PI 10 in Wroclaw). Mean concentration of Cu in all samples were also very high and was ranging from 347 mg/kg in Opole up to 831 mg/kg in Warszawa. Pollution indices PI for Cu were exceeding value of 1 in all cities and were as high as 12.7 in Krakow, 7.7 in Opole, 9.9 in Wroclaw and 18.5 in Warszawa.

Cadmium, copper, zinc, lead and antimony are considered by multiple researchers as an indication of non-exhaust traffic emission (sourced mostly from brake or tire wear). As estimated by Johansson^41^, more than 90% of the road traffic emissions of Cu is caused by brake wear. Moreover when considering ratio Cu/Sb (between 4.6 and 8.9 depending on the authors) as indication of a typical brake wear particle according to authors^42–44^ it can be concluded that Sb and Cu in all investigated cities are primarily sourced from brake lining wear. Cu-Sb ratio in Krakow has equaled 5.62, Warszawa 6.11, Wroclaw 7.38 and in Opole 8.06.

### Human health risk assessment for inhabitants of warszawa, krakow, wroclaw and opole

#### Ingestion pathway

The potential non-carcinogenic human health risk related to ingestion of sidewalk dust by both children and adults (Table 3) are presented in the descending order of deleterious substances**:** Sb > Fe > Co > W > Zr > Pb > Se > As > Al > Cu > Ni > Mn > Zn > Cd > Ba > Mo > Sr > Sn. HQ_ing_ indices calculated for individual metals in sidewalk has not exceeded acceptable level of 1, thus indicating negligible non-carcinogenic toxic risk, except for concentration of antimony in Warszawa’s sidewalk dust, which can pose health risk for children when ingested. Considering an average HQ indices calculated for all cities it can be concluded that the potential non-carcinogenic human health risk is higher for children than it is for adults. Values of hazard quotient (HQ) calculated for children were one magnitude higher than those determined for adults and were as follows: Sb (7.02E-01), Fe (2.99E-01), Co (2.89E-01), W (2.62E-01), Zr (2.48E-01), Pb (2.20E-01), Se (1.68E-01), As (1.06E-01), Al (9.31E-02), Cu (4.52E-02), Ni (3.61E-02), Mn (3.17E-02), Zn (2.23E-02), Cd (1.22E-02), Ba (9.22E-03), Mo (9.07E-03), Mo (9.07E-03), Sr (1.56E-03) and Sn (2.49E-04).

**Table 3:** Non-carcinogenic health risk for ingestion of sidewalk dust by children and adults.

Elements	RfD**	Krakow		Opole		Wroclaw		Warszawa	
	**mg/(kgxday)	ADD*	HQI	ADD*	HQI	ADD*	HQI	ADD*	HQI
**CHILDREN**									
Al	1.00E + 00	9.18E-02	9.18E-02	1.10E-01	1.10E-01	9.00E-02	9.00E-02	8.03E-02	8.03E-02
As	3.00E-04	6.11E-05	2.04E-01	1.98E-05	6.59E-02	2.47E-05	8.22E-02	2.16E-05	7.19E-02
Ba	2.00E-01	1.83E-03	9.16E-03	1.52E-03	7.62E-03	2.04E-03	1.02E-02	1.99E-03	9.93E-03
Cd	1.00E-03	1.65E-05	1.65E-02	6.40E-06	6.40E-03	2.15E-05	2.15E-02	4.35E-06	4.35E-03
Co	3.00E-03	6.99E-05	2.33E-01	9.93E-05	3.31E-01	1.05E-04	3.50E-01	7.30E-05	2.43E-01
Cu	4.00E-02	1.88E-03	4.71E-02	1.14E-03	2.85E-02	1.47E-03	3.68E-02	2.73E-03	6.83E-02
Fe	7.00E-01	1.95E-01	2.79E-01	1.78E-01	2.55E-01	1.95E-01	2.79E-01	2.69E-01	3.84E-01
Mn	1.40E-01	6.08E-03	4.34E-02	3.94E-03	2.81E-02	3.59E-03	2.57E-02	4.15E-03	2.96E-02
Mo	5.00E-03	4.10E-05	8.20E-03	3.12E-05	6.24E-03	3.55E-05	7.10E-03	7.37E-05	1.47E-02
Ni	1.10E-02	2.23E-04	2.03E-02	3.82E-04	3.47E-02	6.78E-04	6.17E-02	3.07E-04	2.79E-02
Pb	3.50E-03	1.02E-03	2.92E-01	7.10E-04	2.03E-01	6.33E-04	1.81E-01	7.10E-04	2.03E-01
Sb	4.00E-04	3.35E-04	8.37E-01	1.41E-04	3.53E-01	1.99E-04	4.98E-01	4.48E-04	1.12E + 00
Se	5.00E-03	8.58E-04	1.72E-01	8.71E-04	1.74E-01	7.25E-04	1.45E-01	9.14E-04	1.83E-01
Sn	6.00E-01	1.40E-04	2.33E-04	1.09E-04	1.81E-04	1.54E-04	2.57E-04	1.96E-04	3.26E-04
Sr	6.00E-01	8.24E-04	1.37E-03	1.06E-03	1.76E-03	1.01E-03	1.69E-03	8.52E-04	1.42E-03
W	8.00E-04	2.66E-04	3.32E-01	1.39E-04	1.74E-01	2.22E-04	2.78E-01	2.12E-04	2.64E-01
Zn	3.00E-01	8.83E-03	2.94E-02	4.38E-03	1.46E-02	7.02E-03	2.34E-02	6.55E-03	2.18E-02
Zr	8.00E-05	2.15E-05	2.69E-01	1.83E-05	2.29E-01	1.96E-05	2.45E-01	1.97E-05	2.47E-01
**HI**_**ing**_			**2.88E + 00**		**2.02E + 00**		**2.34E + 00**		**2.97E + 00**
**ADULTS**									
Al	1.00E + 00	9.84E-03	9.84E-03	1.18E-02	1.18E-02	9.65E-03	9.65E-03	8.61E-03	8.61E-03
As	3.00E-04	6.55E-06	2.18E-02	2.12E-06	7.06E-03	2.64E-06	8.81E-03	2.31E-06	7.71E-03
Ba	2.00E-01	1.96E-04	9.81E-04	1.63E-04	8.16E-04	2.18E-04	1.09E-03	2.13E-04	1.06E-03
Cd	1.00E-03	1.77E-06	1.77E-03	6.86E-07	6.86E-04	2.30E-06	2.30E-03	4.66E-07	4.66E-04
Co	3.00E-03	7.49E-06	2.50E-02	1.06E-05	3.54E-02	1.13E-05	3.75E-02	7.83E-06	2.61E-02
Cu	4.00E-02	2.02E-04	5.05E-03	1.22E-04	3.06E-03	1.58E-04	3.94E-03	2.93E-04	7.32E-03
Fe	7.00E-01	2.09E-02	2.99E-02	1.91E-02	2.73E-02	2.09E-02	2.99E-02	2.88E-02	4.12E-02
Mn	1.40E-01	6.51E-04	4.65E-03	4.22E-04	3.01E-03	3.85E-04	2.75E-03	4.44E-04	3.17E-03
Mo	5.00E-03	4.39E-06	8.78E-04	3.34E-06	6.69E-04	3.80E-06	7.60E-04	7.89E-06	1.58E-03
Ni	1.10E-02	2.39E-05	2.17E-03	4.09E-05	3.72E-03	7.27E-05	6.61E-03	3.29E-05	2.99E-03
Pb	3.50E-03	1.09E-04	3.13E-02	7.60E-05	2.17E-02	6.78E-05	1.94E-02	7.60E-05	2.17E-02
Sb	4.00E-04	3.59E-05	8.97E-02	1.51E-05	3.79E-02	2.14E-05	5.34E-02	4.80E-05	1.20E-01
Se	5.00E-03	9.19E-05	1.84E-02	9.33E-05	1.87E-02	7.77E-05	1.55E-02	9.80E-05	1.96E-02
Sn	6.00E-01	1.50E-05	2.50E-05	1.16E-05	1.94E-05	1.65E-05	2.75E-05	2.10E-05	3.49E-05
Sr	6.00E-01	8.83E-05	1.47E-04	1.13E-04	1.89E-04	1.09E-04	1.81E-04	9.13E-05	1.52E-04
W	8.00E-04	2.85E-05	3.56E-02	1.49E-05	1.86E-02	2.38E-05	2.98E-02	2.27E-05	2.83E-02
Zn	3.00E-01	9.46E-04	3.15E-03	4.69E-04	1.56E-03	7.52E-04	2.51E-03	7.01E-04	2.34E-03
Zr	8.00E-05	2.31E-06	2.88E-02	1.96E-06	2.45E-02	2.10E-06	2.63E-02	2.11E-06	2.64E-02
**HI**_**ing**_			**3.09E-01**		**2.17E-01**		**2.50E-01**		**3.19E-01**

*ADD (average daily dose through ingestion), **RfD (mg/kg body weight per day), HQI (unitless).

The sum of individual HQs, which determines hazard index (HI) has exceeded safe threshold value of 1 in all cities for both children and adults, thus indicating potential non-carcinogenic effect of ingestion of sidewalk dust. The values of HI indices were the highest in most congested cities, and they were as follows: in Warszawa (HI 2.97 for children and 0.319 for adults), in Krakow (HI 2.88 for children and 0.309 for adults), in Wroclaw (HI 2.34 for children and 0.25 for adults) and in Opole (HI 2.02 for children and 0.217 for adults).

Carcinogenic risk (CR) calculated for As, Cd, Ni and Pb in sidewalk dust (Table 4) has exceeded safe level of 1 × 10^-4^ in all cities except for Warszawa thus indicating that potential carcinogenic risk posed by those metals to children via ingestion occurs and it is not negligible.

**Table 4:** Carcinogenic health risk for ingestion of sidewalk dust by children and adults.

Elements	SF	Krakow	Opole	Wroclaw	Warszawa
	(mg/kg body per day)^−1^	CR_ing_			
**CHILDREN**					
As	1.20E + 01	7.33E-04	1.65E-06	2.05E-06	1.80E-06
Cd	1.50E + 01	2.48E-04	9.60E-05	3.22E-04	6.53E-05
Ni	9.00E-01	2.01E-04	3.44E-04	6.11E-04	2.76E-04
Pb	4.20E-02	4.29E-05	2.98E-05	2.66E-05	2.98E-05
∑ **CR**_**ing**_		**3.06E-04**	**1.18E-04**	**2.40E-04**	**9.33E-05**
**ADULTS**					
As	1.20E + 01	5.46E-07	1.77E-07	2.20E-07	1.93E-07
Cd	1.50E + 01	2.66E-05	1.03E-05	2.13E-05	6.99E-06
Ni	9.00E-01	2.15E-05	3.68E-05	6.54E-05	2.96E-05
Pb	4.20E-02	4.60E-06	3.19E-06	2.85E-06	3.19E-06
∑ **CR**_**ing**_		**1.33E-05**	**1.26E-05**	**2.24E-05**	**9.99E-06**

Furthermore carcinogenic health risk associated with the ingestion of sidewalk dust by adults was not found (Table 4). Values of CR calculated for As, Cd, Ni and Pb has not exceeded safe acceptable range of 1 × 10^-6^-1 × 10^-4^.

#### Dermal contact pathway

The results presented in Table 5 has revealed a greater risk associated with dermal contact with sidewalk dust for adults rather than for children. These findings are consistent with the results of other researchers^34,45^. The highest HI was determined for adults exposed to sidewalk dust via dermal contact in Wroclaw (1.19), Krakow (0.95), Opole (0.389) and Warszawa (0.284). Only in Wroclaw HI_dermal_ has exceeded acceptable and safe level of 1, while in other cites values of hazard indicies were below unity. Values of HI_dermal_ calculated for children were about ten times smaller than those determined for adults and were as follows: 0.167 in Wroclaw, 0.133 in Krakow, 0.0399 in Warszawa and 0.0546 in Opole respectively.

**Table 5:** Non-carcinogenic health risk associated with dermal contact with sidewalk dust by children and adults.

Elements	GIABS	RfD_derm_**	Krakow		Opole		Wroclaw		Warszawa	
		**mg/(kgxday)	ADD*	HQI	ADD*	HQI	ADD*	HQI	ADD*	HQI
**CHILDREN**										
Cd	2.50E-02	2.50E-05	7.72E-08	1.23E-01	2.99E-08	4.78E-02	1.00E-07	1.60E-01	2.03E-08	3.25E-02
Cu	1,00E + 00	1.20E-02	8.80E-06	7.33E-04	5.33E-06	4.44E-04	6.87E-06	5.73E-04	1.28E-05	1.06E-03
Pb	1,00E + 00	5.25E-04	4.77E-06	9.08E-03	3.31E-06	6.31E-03	2.95E-06	5.63E-03	3.31E-06	6.31E-03
Zn	1,00E + 00	6.00E-01	4.12E-05	6.87E-05	2.04E-05	3.40E-05	3.28E-05	5.46E-05	3.06E-05	5.09E-05
HI_derm_			**1.33E-01**		**5.46E-02**			**1.67E-01**		**3.99E-02**
**ADULTS**										
Cd	2.50E-02	2.50E-05	5.50E-07	8.80E-01	2.13E-07	3.40E-01	7.15E-07	1.14E + 00	1.45E-07	2.31E-01
Cu	1,00E + 00	1.20E-02	6.27E-05	5.22E-03	3.80E-05	3.16E-03	4.90E-05	4.08E-03	9.09E-05	7.57E-03
Pb	1,00E + 00	5.25E-04	3.40E-05	6.47E-02	2.36E-05	4.49E-02	2.10E-05	4.01E-02	2.36E-05	4.49E-02
Zn	1,00E + 00	6.00E-01	2.94E-04	4.89E-04	1.46E-04	2.43E-04	2.33E-04	3.89E-04	2.18E-04	3.63E-04
**HI**_**dermal**_				**9.50E-01**		**3.89E-01**		**1.19E + 00**		**2.84E-01**

^*^ADD (average daily dose via dermal contact). **RfD_derm_ (mg/kg body weight per day). HQI (unitless).

The potential non-carcinogenic human health risk related to dermal contact with individual deleterious substances in sidewalk dust for both children and adults are presented in the descending order: Cd > Pb > Cu > Zn.

Furthermore, no carcinogenic health risk associated with sidewalk dust dermal contact for children was found, as the CR values for As and Pb did not exceed acceptable level of 1 × 10^−4^ (Table 6). Carcinogenic risk was however significant for adults, since CR values calculated for As and Pb were 3.04 E-04.

**Table 6:** Carcinogenic health risk associated with dermal contact with sidewalk dust by children and adults.

Elements	GIABS	SF	Krakow	Opole	Wroclaw	Warszawa
		(mg/kg body per day)^-1^				
**CHILDREN**						
As	1.00E + 01	1.20E + 01	3.42E-06	1.11E-06	1.38E-06	1.21E-06
Pb	1.00E + 01	4.20E-02	1.14E-04	7.88E-05	7.03E-05	7.89E-05
∑ **CR**_**dermal**_			**5.85E-05**	**4.00E-05**	**3.59E-05**	**4.00E-05**
**ADULTS**						
As	1.00E + 01	1.20E + 01	1.69E-07	5.48E-08	6.83E-08	5.98E-08
Pb	1.00E + 01	4.20E-02	8.09E-04	5.62E-04	5.01E-04	5.62E-04
∑ **CR**_**dermal**_			**4.04E-04**	**2.81E-04**	**2.51E-04**	**2.81E-04**

#### Inhalation pathway

The potential non-carcinogenic human health risk related to the inhalation of sidewalk dust for both children and adults are presented in the descending order of individual deleterious substances: Mn > Ni > Co > Ba > As > Cd > Se. Values of Hazardous Indices (HI_inh_) determined for sidewalk dust in all four cities were below the unity (Table 7), thus indicating negligible potential non-carcinogenic health human risk associated with the inhalation of sidewalk dust by both children and adults.

**Table 7:** Non-carcinogenic health risk for the inhalation of sidewalk dust by children and adults.

Elements	RfD_inh_**		Krakow		Opole	Wroclaw		Warszawa	
	mg/m^3^	ADD_inh_*	HQI	ADD_inh_*	HQI	ADD_inh_*	HQI	ADD_inh_*	HQI
**CHILDREN**									
Al	—	6.75E-06	—	8.1E-06		6.62E-06		5.91E-06	
As	1.50E-05	4.49E-09	3.00E-07	1.45E-09	9.7E-08	1.81E-09	1.21E-07	1.59E-09	1.06E-07
Ba	5.00E-04	1.35E-07	2.69E-07	1.12E-07	2.24E-07	1.5E-07	3E-07	1.46E-07	2.92E-07
Cd	1.00E-05	1.22E-09	1.22E-07	4.71E-10	4.71E-08	1.58E-09	1.58E-07	3.2E-10	3.20E-08
Co	6.00E-06	5.14E-09	8.57E-07	7.3E-09	1.22E-06	7.73E-09	1.29E-06	5.37E-09	8.95E-07
Cu	—	1.39E-07	—	8.39E-08	—	1.08E-07	—	2.01E-07	—
Fe	—	1.43E-05	—	1.31E-05	—	1.44E-05	—	1.98E-05	—
Mn	5.00E-05	4.47E-07	8.93E-06	2.9E-07	5.79E-06	2.64E-07	5.28E-06	3.05E-07	6.10E-06
Mo	—	3.01E-09	—	2.3E-09	—	2.61E-09	—	5.42E-09	—
Ni	1.40E-05	1.64E-08	1.17E-06	2.81E-08	2E-06	4.99E-08	3.56E-06	2.26E-08	1.61E-06
Pb	—	7.51E-08	—	5.22E-08	—	4.65E-08	—	5.22E-08	—
Sb	—	2.46E-08	—	1.04E-08	—	1.47E-08	—	3.29E-08	—
Se	2.00E-02	6.31E-08	3.15E-09	6.41E-08	3.2E-09	5.33E-08	2.67E-09	6.72E-08	3.36E-09
Sn	—	1.03E-08	—	7.99E-09	—	1.13E-08	—	1.44E-08	—
Sr	—	6.06E-08	—	7.79E-08	—	7.46E-08	—	6.27E-08	—
W	—	1.95E-08	—	1.02E-08	—	1.63E-08	—	1.56E-08	—
Zn	—	6.49E-07	—	3.22E-07	—	5.16E-07	—	4.81E-07	—
Zr	—	1.58E-09	—	1.35E-09	—	1.44E-09	—	1.45E-09	—
HI_inh_			**1.17E-05**		**9.39E-06**		**1.07E-05**		**9.04E-06**
**ADULTS**									
Al	—	2.89E-06	—	3.47E-06	—	2.84E-06	—	2.53E-06	—
As	1.50E-05	1.93E-09	1,28E-07	6.23E-10	4.16E-08	7.77E-10	5.18E-08	6.80E-10	4.53E-08
Ba	5.00E-04	5.77E-08	1,15E-07	4.80E-08	9.60E-08	6.42E-08	1.28E-07	6.26E-08	1.25E-07
Cd	1.00E-05	5.21E-10	5,21E-08	2.02E-10	2.02E-08	6.77E-10	6.77E-08	1.37E-10	1.37E-08
Co	6.00E-06	2.20E-09	3,67E-07	3.13E-09	5.21E-07	3.31E-09	5.52E-07	2.30E-09	3.84E-07
Cu	—	5.94E-08	—	3.60E-08	—	4.64E-08	—	8.61E-08	—
Fe	—	6.15E-06	—	5.62E-06	—	6.15E-06	—	8.48E-06	—
Mn	5.00E-05	1.91E-07	3,83E-06	1.24E-07	2.48E-06	1.13E-07	2.26E-06	1.31E-07	2.61E-06
Mo	—	1.29E-09	—	9.84E-10	—	1.12E-09	—	2.32E-09	—
Ni	1.40E-05	7.03E-09	5,02E-07	1.20E-08	8.59E-07	2.14E-08	1.53E-06	9.67E-09	6.91E-07
Pb	—	3.22E-08	—	2.24E-08	—	1.99E-08	—	2.24E-08	—
Sb	—	1.06E-08	—	4.45E-09	—	6.28E-09	—	1.41E-08	—
Se	2.00E-02	2.70E-08	1,35E-09	2.75E-08	1.37E-09	2.29E-08	1.14E-09	2.88E-08	1.44E-09
Sn	—	4.41E-09	—	3.42E-09	—	4.85E-09	—	6.16E-09	—
Sr	—	2.60E-08	—	3.34E-08	—	3.20E-08	—	2.69E-08	—
W	—	8.37E-09	—	4.38E-09	—	7.00E-09	—	6.67E-09	—
Zn	—	2.78E-07	—	1.38E-07	—	2.21E-07	—	2.06E-07	—
Zr	—	6.78E-10	—	5.77E-10	—	6.18E-10	—	6.22E-10	—
**HI**_**inh**_			**5,00E-06**		**4.02E-06**		**4.59E-06**		**3.87E-06**

^*^ADD (average daily dose through inhalation). **RfD_inh_ (mg/m^3^). HQI (unitless).

It was found that non-carcinogenic risk associated with the inhalation of sidewalk dust is two times higher for children than it is to adults due to the fact that kids are introducing 50% more air into their lungs per body mass and moreover their respiratory systems are not entirely developed yet so they are more easily to be damaged^45^.

Consequently no carcinogenic health risk associated with the inhalation of sidewalk dust was found for children and adults (Table 8), as the CR values for As, Cd, Ni and Pb did not exceed acceptable treshold level of 10 ^-4^. Carcinogenic health risk was however higher for children than for adults, but in both cases was considered as negligible.

**Table 8:** Carcinogenic health risk for the inhalation of sidewalk dust by children and adults.

Elements	IUR	Krakow	Opole	Wroclaw	Warszawa
	(µg/m^−3^)^−1^	CR_inh_			
**CHILDREN**					
As	4.30E-03	1.93E-11	6.25E-12	7.8E-12	6.82E-12
Cd	1.80E-03	2.19E-12	8.47E-13	2.84E-12	3.2E-10
Ni	2.40E-04	1.64E-08	2.81E-08	4.99E-08	2.26E-08
Pb	1.20E-05	7.51E-08	5.22E-08	4.65E-08	5.22E-08
∑ **CR**_**inh**_		**2.29E-08**	**2.01E-08**	**2.41E-08**	**1.88E-08**
**ADULTS**					
As	4.30E-03	8.28E-12	2.68E-12	3.34E-12	2.92E-12
Cd	1.80E-03	9.38E-13	3.63E-13	1.22E-12	2.47E-13
Ni	2.40E-04	1.69E-12	2.89E-12	5.13E-12	2.32E-12
Pb	1.20E-05	3.86E-13	2.68E-13	2.39E-13	2.68E-13
∑ **Cr**_**inh**_		**2.82E-12**	**1.55E-12**	**2.48E-12**	**1.44E-12**

## Discussion

Sidewalk dust collected from four big cities of Poland is heavily contaminated with all of the investigated metals, especially with antimony, selenium, cadmium, copper, zinc, lead. An average concentration of Se in sidewalk dust from all cities was as high as 256 [mg/kg], Sb 102 [mg/kg], Zn 2036 [mg/kg], Cd 3.71 [mg/kg], Cu 550 [mg/kg] and Pb 234 [mg/kg]. Concentration of metals in sidewalk dust has greatly exceeded background values on average 256 times with respect to Se, 68 times with respect to Sb, 21 times with respect to Zn, 12 times with respect to Cu, Pb and Cd. As indicated by many authors metals such as Zn, Cd, Cu, Pb in road or sidewalk dust are mostly derived from traffic related sources, such as non-exhaust traffic emission from brake, tire or clutch wear, other vehicle and road component degradation^46^, and/or from exhaust emission systems as a byproduct of combustion of different types of fuel (gasoline or diesel)^47^. Moreover XRD and SEM-EDS analysis revealed quartz and aluminum silicates, as the main constituents of sidewalk dust, of probable geogenic origin. However diffractograms as well as SEM images depicted also the presence of titanates, Ag as well as REE (such as La, Ce, Nd and Sm), as secondary constituents of sidewalk dust, of probable anthropogenic origin. These results are consistent with the finding of other authors, which has reported that Platinum Group Elements (PGE) such as Pt, Pd, Ir, other noble metals (Ag, Au) as well as some Rare Earth Elements (REE) in road or sidewalk dust can originate from exhaust catalytic converters or can be sourced from various components of hybrid electric vehicles (HEVs)^47–49^, since lanthanum is used in batteries, catalyst and lenses, cerium is currently used as fuel additive, catalyst or optical polish, neodymium and samarium are commonly used in lasers, magnets or in car computers and LCD screens^40^.

It can be concluded that the exposition to sidewalk dust via all pathways is especially dangerous in heavily congested urban areas, in close vicinity to traffic lights (thus confirming findings of previous researches^50^.

Hazardous indices determined for various exposure pathways for inhabitants of Warszawa, Krakow, Wroclaw and Opole indicate that the greatest health risk is associated with the introduction of heavy metals from sidewalk dust through ingestion. Ingestion is the primary pathway which has the most harmful effect on both children and the adults when compared to other exposure pathways like dermal and inhalation.

Non-carcinogenic risk associated with the ingestion of sidewalk dust by children was two orders of magnitude higher than the corresponding dermal risk and was four to five orders of magnitude higher than inhalation value. These findings are consistent with the reports of other, multiple researchers^32,34,45^. For adults non-carcinogenic risk associated with the ingestion of sidewalk dust is comparable with dermal contact risk and five orders of magnitude higher when compared to the risk of dust inhalation.

Children are especially exposed to metals in sidewalk dust due to the fact that on average, the dose introduced into the child’s body by ingestion is nearly ten times greater than that of an adult. The hazards indices (HI) calculated for the following metals (Ba, Cd, Co, Cu, Mn, Mo, Pb, Sb, Se, Sn, Zn and Zr) for the ingestion of sidewalk dust by children has significantly exceeded safe level of 1 (from 4.1 in Opole to 6.03 in Warszawa) in all cities, thus confirming that the exposure to sidewalk dust can cause potential health hazard to children.

Values of hazard indices determined for adults revealed potential non-carcinogenic risk for sidewalk dust ingestion only for Krakow inhabitants (HI 2.37). The total non- carcinogenic risk calculated as the sum of HI values for the individual exposure pathways was as high as 2.59 with respect to children and 0.524 with respect to adults. Therefore it can be concluded that the sidewalk dust can cause a higher potential health risk for children than for adults. Especially concern should be paid to the contamination of sidewalk dust with Cd, Zn since those elements are the most mobile and consequently bioavailable.

The results of human health risk assessment related to sidewalk dust exposure may confirm the purposefulness of creating areas of limited traffic in the city centers, where pedestrians, tourists prevails and they are potentially exposed to the risk of ingestion, dermal contact or inhalation of sidewalk dust.

## References

[1] Maynard D, Coull BA, Gryparis A, Schwartz J (2007). Mortality Risk Associated With Short-Term Exposure to Traffic Particles and Sulfates. Environ. Health Perspect..

[2] Meister K, Johansson C, Forsberg B (2012). Estimated short-term effects of coarse particles on daily mortality in Stockholm, Sweden. Environ. Health Perspect..

[3] EEA Report No. 28/2016, Air Quality in Europe — 2016 Report.

[4] EEA Report No. 9/2013, Air Quality in Europe — 2013 Report.

[5] Amato F (2014). Urban air quality: The challenge of traffic non-exhaust emissions. J Hazard Mater..

[6] Pope AC, Dockery DW (2006). Health Effects of Fine Particulate Air Pollution: Lines that Connect. J. Air Waste Manag. Assoc..

[7] Perez L (2009). Size Fractionate Particulate Matter, Vehicle Traffic, and Case-Specific Daily Mortality in Barcelona, Spain. Environ. Sci. Technol..

[8] Qiu H (2012). Effects of coarse particulate matter on emergency hospital admissions for respiratory diseases: A time-series analysis in Hong Kong. Environ. Health Perspect..

[9] Loeb LA (2001). A mutator phenotype in cancer. Cancer Res..

[10] Potgieter-Vermaak S, Rotondo G, Novakovic V, Rollins S, van Grieken R (2012). Component−specific toxic concerns of the inhalable fraction of urban road dust. Environ. Geochem. Hlth..

[11] WHO Report. World Health Organization (WHO) Regional Office for Europe. Health effects of particulate matter: Policy implications for countries in Eastern Europe, Caucasus and Central Asia, http://www.euro.who.int/__data/assets/pdf_file/0006/189051/Health-effects-of-particulate-matter-final-Eng.pdf (access 23.12.2016) (2013).

[12] Barlow, T. J *et al*. Non-exhaust particulate matter emissions from road traffic: Summary report. TRL report for DEFRA. Scottish Executive. Welsh Assembly Government. DoENI (2007).

[13] Querol X (2007). Source origin of trace elements in PM from regional background, urban and industrial sites of Spain. Atmos. Environ.

[14] Thorpe A, Harrison RM (2008). Sources and properties of non-exhaust particulate matter from road traffic: a review. Sci Total Environ.

[15] McKenzie ER, Money JE, Green PG, Young TM (2009). Metals associated with stormwater-relevant brake and tire samples. Sci. Total Environ..

[16] Carrero JA, Goienaga N, Olivares M, Martinez-Arkarazo I, Arana G (2012). Raman spectroscopy assisted with XRF and chemical simulation to assess the synergic impacts of guardrails and traffic pollutants on urban soils. J. Raman Spectrosc..

[17] Pant P, Harrison RM (2013). Estimation of the contribution of road traffic emissions to particulate matter concentrations from field measurements: A review. Atmos. Environ..

[18] GUS Statistic Office Poland, Environmental Protection - Environment. stat.gov.pl/download/gfx/portalinformacyjny/pl/…/1/…/ochrona_srodowiska_2016.pdf (access 6.08.2017) (2016).

[19] Rexeis M, Hausberger S (2009). Trend of vehicle emission levels until 2020-Prognosis based on current vehicle measurements and future emission legislation. Atmos. Environ..

[20] Bukowiecki, N. *et al*. PM10 emission factors of abrasion particles from road traffic (APART). Swiss Association of Road and Transportation Experts (VSS) (2009).

[21] van der Gon HA (2013). The policy relevance of wear emissions from road transport, now and in the future—an international workshop report and consensus statement. J. Air Waste Manag. Assoc..

[22] Garg BD, Cadle SH, Mulawa PA, Groblicki PJ (2000). Brake wear particulate matter emissions. Environ. Sci. Technol..

[23] Adamiec E (2017). Chemical fractionation and mobility of traffic related elements in road environments. Environ. Geochem. Health.

[24] Kukutschová J (2011). On airborne nano/micro-sized wear particles released from low-metallic automotive brakes. Environ. Pollut..

[25] Adamiec E (2017). Road Environments: Impact of Metals on Human Health in Heavily Congested Cities of Poland. Int J Environ Res Public Health.

[26] Ewen C, Anagnostopoulou MA, Ward NL (2009). Monitoring of heavy metal levels in roadside dusts of Thessaloniki, Greece in relation to motor vehicle traffic density and flow. Environ, Monit. Assess..

[27] Environmental Protection Agency. Method 3050A: Acid Digestion of Sediments, Sludges, and Soils, Revision 1; EPA: Washington, DC, USA (1992).

[28] Environmental Protection Agency. Method 6020B: Inductively Coupled Plasma-Mass Spectrometry, Revision 2; EPA: Washington, DC, USA (1998).

[29] Turekian, K. K. & Wedephol, H. H. Geological Society of America Bulletin, **72**, 175–192 (1961).

[30] U.S. Environmental Protection Agency. Exposure Factors Handbook (Final), ed.; EPA/600/R-09/052F; gov/docs/ML1400/ML14007A666.pdf (accessed on 27 January 2017) (2011).

[31] Regional Screening Levels (RSLs)—Generic Tables (May 2016). U.S. Environmental Protection Agency. Available online, https://www.epa.gov/risk/regional-screening-levels-rsls-generic-tables-may-2016, (access on 22 January 2017) (2016).

[32] Du Y, Gaoa B, Zhoua H, Jua X, Haoa H, Yina S (2013). Health Risk Assessment of Heavy Metals in Road Dusts in Urban Parks of Beijing, China. Procedia. Environ. Sci.

[33] Yu, B., Wang Y. & Zhou Q., Human Health Risk Assessment Based on Toxicity Characteristic Leaching Procedure and Simple Bioaccessiblility Extraction Test of Toxic Metals in Urban Street Dust of Tianjin, China. *PLoS ONE* **9** (3). (2014). 10.1371/journal.pone.0092459 PMC3961371 24651129

[34] Zhou Q (2015). Residents health risk of Pb, Cd and Cu exposure to street dust based on different particle sizes around zinc smelting plant Northeast of China. Environ. Geochem. Health..

[35] U.S. Environmental Protection Agency. Exposure Factors Handbook 2011 Edition (Final)., Washington, DC, EPA/600/R-09/052F, https://www.nrc.gov/docs/ML1400/ML14007A666.pdf., (access 27.01.2017) (2011).

[36] Gruszecka-Kosowska A (2018). Assessment of the Krakow inhabitants’ health risk caused by the exposure to inhalation of outdoor air contaminants. Stoch Environ Res Risk Assess.

[37] U.S. Environmental Protection Agency. Risk assessment guidance for superfund. Vol. 1: Human health evaluation manual. Part A. Interim final. Office of Emergency and Remedial Response, US EPA, Washington, DC, USA (1989).

[38] U.S. Environmental Protection Agency. Child-specific exposure factors handbook. EPA-600-P-00-002B. National Center for Environmental Assessment (2002).

[39] Hjortenkrans DST, Bergbäck BG, Häggerud AV (2007). Metal emissions from brake linings and tires: case studies of Stockholm, Sweden 1995/1998 and 2005. Environ. Sci. Technol..

[40] Yano J, Takanori M, Shin-ichi S (2016). Rare earth element recovery potentials from end-of-life hybrid electric vehicle components in 2010–2030. J Mater Cycles Waste Manag.

[41] Johansson Ch., B. L. & Forsberg B. The effects of congestions tax on air quality and health. *Atmos. Environ* **43** (31) (2009).

[42] Sternbeck J, Sjodin A, Andreasson K (2002). Metal emissions from road traffic and the influence of resuspension - results from two tunnel studies. Atmos. Environ..

[43] Alves CA, Oliveira C, Martins N, Caseiro A, Pio C, Matos M, Silva HF (2016). Camões Road tunnel, roadside and measurement of aliphatic compounds in size-segregated particulate matter. Atmos. Res..

[44] Amato F (2009). Spatial and chemical patterns of PM10 in road dust deposited in urban environment. Atmos. Environ..

[45] Trojanowska, M. & Świetlik, R. Ocena narażenia mieszkańców miast na metale ciężkie obecne w pyłach ulicznych. *Autobusy* **12**, 474–478 (in Polish) (2016).

[46] Adamiec E, Jarosz-Krzemińska E, Wieszała R (2016). Heavy metals from non-exhaust vehicle emissions in urban and motorway road dusts. Environ Monit. Assess..

[47] Golokhvast KS (2015). Size-segregated emissions and metal content of vehicle-emitted particles as a function of mileage: Implications to population exposure. Env. Reseach.

[48] Limbeck A, Puls C, Handler M (2007). Platinum and Palladium Emissions from On-Road Vehicles in the Kaisermühlen Tunnel (Vienna, Austria). Environ. Sci. Technol..

[49] Wiseman CLS (2018). An assessment of the inhalation bioaccessibility of platinum group elements in road dust using a simulated lung fluid. Environ Pollut..

[50] Taiwo AM (2017). Assessment of health risks associated with road dust in major hotspots in Abeokuta metropolis, Ogun state, southwestern Nigeria. Stoch Environ Res Risk Assess.

